# Solar Light Induced Photon-Assisted Synthesis of TiO_2_ Supported Highly Dispersed Ru Nanoparticle Catalysts

**DOI:** 10.3390/ma11112329

**Published:** 2018-11-19

**Authors:** Joanna Wojciechowska, Elisa Gitzhofer, Jacek Grams, Agnieszka M. Ruppert, Nicolas Keller

**Affiliations:** 1 Institute of General and Ecological Chemistry, Faculty of Chemistry, Lodz University of Technology, ul. Żeromskiego 116, 90-924 Łódź, Poland; joanna.wojciechowska@etu.unistra.fr (J.W.); jacek.grams@p.lodz.pl (J.G.); agnieszka.ruppert@p.lodz.pl (A.M.R.); 2 Institut de Chimie et Procédés pour l’Energie, l’Environnement et la Santé, CNRS/University of Strasbourg, 25 rue Becquerel, 67087 Strasbourg, France; elisagitz@gmail.com

**Keywords:** Ru/TiO_2_ catalyst, catalyst preparation, sub-nanometric particle size distribution, highly dispersed Ru nanoparticle, reaction mechanism, photodeposition, photon-assisted synthesis

## Abstract

Ru/TiO_2_ are promising heterogeneous catalysts in different key-reactions taking place in the catalytic conversion of biomass towards fuel additives, biofuels, or biochemicals. TiO_2_ supported highly dispersed nanometric-size metallic Ru catalysts were prepared at room temperature via a solar light induced photon-assisted one-step synthesis in liquid phase, far smaller Ru nanoparticles with sharper size distribution being synthesized when compared to the catalysts that were prepared by impregnation with thermal reduction in hydrogen. The underlying strategy is based on the redox photoactivity of the TiO_2_ semi-conductor support under solar light for allowing the reduction of metal ions pre-adsorbed at the host surface by photogenerated electrons from the conduction band of the semi-conductor in order to get a fine control in terms of size distribution and dispersion, with no need of chemical reductant, final thermal treatment, or external hydrogen. Whether acetylacetonate or chloride was used as precursor, 0.6 nm sub-nanometric metallic Ru particles were synthesized on TiO_2_ with a sharp size distribution at a low loading of 0.5 wt.%. Using the chloride precursor was necessary for preparing Ru/TiO_2_ catalysts with a 0.8 nm sub-nanometric mean particle size at 5 wt.% loading, achieved in basic conditions for benefitting from the enhanced adsorption between the positively-charged chloro-complexes and the negatively-charged TiO_2_ surface. Remarkably, within the 0.5–5 wt.% range, the Ru content had only a slight influence on the sub-nanometric particle size distribution, thanks to the implementation of suitable photo-assisted synthesis conditions. We demonstrated further that a fine control of the metal Ru nanoparticle size on the TiO_2_ support was possible via a controlled nanocluster growth under irradiation, while the nanoparticles revealed a good resistance to thermal sintering.

## 1. Introduction

The crucial role that is played by heterogeneous catalysis in industrial chemical processes usually requires the engineering of tailored supported metal nanoparticles as catalysts, since the catalytic activity and the reaction rates can be strongly influenced by both the shape and the size of supported metallic nanoparticles [1,2,3]. A fine control over both the size and the size distribution of supported metal nanoparticles is necessary for increasing the metal dispersion for a given metal loading and improving the performances of catalytic reactions, as well as for investigating the mechanisms taking place in size–dependent catalytic reactions thanks to the establishment of more detailed structure-properties relationship studies [4,5,6].

The solar light photon-assisted synthesis method is considered as a sustainable and elegant preparation method for synthesising well-defined and small-size supported metal nanoparticles, and for providing a control over their size, distribution, and oxidation state [7]. Provided that the support carrier is a semi-conductor material, the underlying strategy relies on the use of the redox photo-activity of the semi-conductor support for promoting under appropriate light electrons from the valence to the conduction band of the semi-conductor, subsequently available for reducing metal ions adsorbed at the support surface. The low-temperature and one-step photon-assisted synthesis method differs from the most widely used preparation methods, for which a final post-deposition reduction/activation/ step is required to form the supported metallic particles after the metal precursor has been introduced onto the support carrier, and usually consisting either in a thermal treatment with the use of external hydrogen, or in a chemical reduction in solution with external reducing agent [8].

Although the influence of the synthesis parameters on the deposition rate and on the metallic nanoparticle shape and it was investigated in some early studies [9], the photodeposition synthesis method has been mainly used for preparing catalytic and photocatalytic materials consisting in monometallic and more scarcely bimetallic particles being dispersed on a medium or high surface area support. TiO_2_ was the main semi-conductor support used, although other materials, such as BiVO_4_ or GaN:ZnO, were also investigated, while Au [10,11], Pd [12], Ag [13,14], Pt [9,15], Rh [16], Cu [17,18], and Pt-Ag [19] were the most studied metals, using usually acetylacetonates, chlorides, nitrates, and chloric acids as metallic precursors.

The aim of this paper is to report on the use of a photodeposition method as an alternative method to classical wet impregnation, followed by a final reduction in temperature under hydrogen for preparing Ru/TiO_2_ catalysts. Indeed, Ru/TiO_2_ are promising heterogeneous catalysts in different key-reactions taking place in the catalytic conversion of biomass towards biochemicals, including e.g., biofuels or fuel additives. Catalysts that were supported on TiO_2_ were proved to be remarkably stable for biomass conversion reactions, while ruthenium is a metal of choice notably for hydrogenating biomass-derived molecules [20]. Till now, the synthesis of Ru nanoparticles at the surface of a support via a photodeposition method remained scarce, and was achieved using (NH_4_)_3_RuCl_6_ or RuCl_3_ as ruthenium salts, and TiO_2_, CdS, CeO_2_ and CuInS_2_ quantum dots as host semiconductors under ultra-violet/vis or visible light irradiation [21,22,23,24].

Here, we show that a one-step photon-assisted synthesis method can be implemented under simulated solar light for preparing Ru/TiO_2_ catalysts with a Ru content ranging from 0.5 to 5 wt.%, with a sharp, nanometer-size and finely tunable Ru particle size distribution.

## 2. Materials and Methods

### 2.1. Ru/TiO_2_ Material Preparation

Aeroxide© P25 TiO_2_ (Evonik, Essen, Germany) has been used as TiO_2_ support for preparing Ru/TiO_2_ catalysts under simulated solar light irradiation. Ruthenium (III) acetylacetonate (Ru(acac)_3_, 97%, Sigma-Aldrich, Saint-Louis, MO, USA), and ruthenium (III) chloride hydrate (RuCl_3_•xH_2_O, min 40% Ru content, Sigma-Aldrich) were used as ruthenium metallic precursors. Dissolution of the Ru(acac)_3_ precursor was achieved in distilled water under stirring at 50 °C for two days, whereas the RuCl_3_ precursor was dissolved under stirring in 10 mL of methanol for 12 h, prior to the addition of 90 mL of distilled water to give a methanol:water ratio of 1:9 *v*/*v*. In each experiment, the TiO_2_ support was dispersed under stirring in 100 mL of ruthenium solution in a beaker-type glass reactor at a 1 g/L concentration, with a precursor concentration depending on the targeted Ru content to be achieved in the final Ru/TiO_2_ material. Prior to irradiation, the suspension was stirred in the dark for 2 h to ensure the establishment of the adsorption-desorption equilibrium. In the case of Ru(acac)_3_, the pH value of the suspension was adjusted with NaOH to pH = 10, whereas for higher Ru loading (5%) using RuCl_3_, the pH was adjusted with NaOH to pH = 8. The TiO_2_ suspension was further exposed under stirring to a 500 W/m^2^ solar light irradiation within an ATLAS Suntest XLS+ reaction chamber equipped with a Xenon arc lamp NXE 2201 (Atlas Material Testing Technology, Mount Prospect, IL, USA).

At each time interval, 1 mL of solution was sampled and filtrated through a 0.20 µm porosity filter (Aireka Scientific Co., Ltd., Hangzhou, China) to remove the titania powder, if any. The deposition was followed by UV-vis spectrophotometry using a Cary 100 Scan Varian spectrophotometer (Agilent Technologies, Santa Clara, CA, USA) monitoring the disappearance of the main absorption peak at λ = 272 nm and λ = 324 nm for Ru(acac)_3_ and RuCl_3_ precursors, respectively. After completion of the process, the catalysts were recovered by filtration and dried at 100 °C for 1 h.

### 2.2. Characterisations

The Ru content in the catalysts was determined by chemical analysis after a microwave-assisted acidic dissolution in aqua regia at 185 °C under autogenic pressure. Inductively coupled plasma optical emission spectroscopy (ICP-OES) was carried out on an Optima 7000 DV spectrometer (Perkin Elmer, Waltham, MA, USA) at the Analysis Platform of IPHC, Strasbourg, France.

The Ru nanoparticle size distribution of Ru/TiO_2_ samples was determined by transmission electron microscopy (TEM) performed using a JEOL 2100F (JEOL Ltd., Tokyo, Japan) with a point resolution of 0.2 nm. The samples were sonically dispersed in an ethanol solution before a drop of the solution was deposited onto a copper grid covered by a holey carbon membrane for observation. The size distributions were calculated for each sample by averaging 300 particles from the TEM images using ImageJ software (National Institutes of Health, Bethesda, MD, USA).

X-Ray Photoelectron Spectroscopy (XPS) characterization was performed on a ThermoVGMultilabESCA3000 spectrometer (Thermo Fisher Scientific, Waltham, MA, USA) that was equipped with an Al K_α_ anode (hλ = 1486.6 eV). The energy shift due to electrostatic charging was subtracted using the adventious sp^2^ carbon C 1s band at 284.6 eV. The spectra were decomposed assuming several contributions, each of them having a Doniach–Sunjic shape [25] and a’ S-shaped’ Shirley type background [26]. Surface atomic ratios were derived using the appropriate experimental sensitivity factors [27].

Thermogravimetric analysis (TGA) was carried out with a 20% (*v*/*v*) O_2_/N_2_ mixture at a flow rate of 40 mL/min at a heating rate of 10 °C/min from 25 °C to 600 °C using a Q 5000 thermoanalyzer from TA instrument (New Castle, DE, USA).

## 3. Results and Discussion

### 3.1. Influence of the Ru Metallic Precursor and of the Targeted Ru Content

Ru(acac)_3_ and hydrated RuCl_3_ are the most used Ru precursors allowing for the preparation of supported Ru catalysts on a wide variety of supports via wet or wetness impregnation with final thermal reduction under hydrogen, so that they both have been used as metallic precursors for preparing a series of ruthenium catalysts with a targeted nominal Ru content from 0.5 wt.% to 5 wt.%.

**Figure 1:**
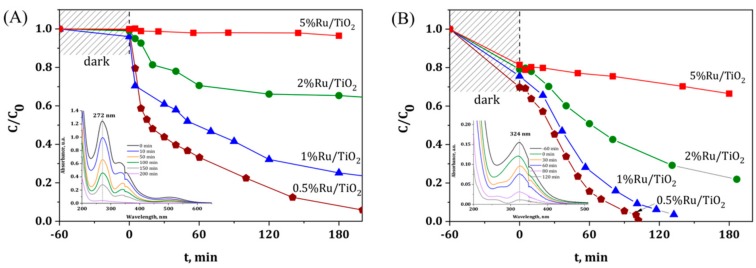
Disappearance of the Ru precursor in the presence of TiO_2_-P25 as a function of the illumination time for (**A**) Ru(acac)_3_ and (**B**) RuCl_3_ precursors, with a Ru concentration ranging from 0.5 to 5 wt.%. Inset: UV-vis absorbance spectra evolution as a function of time during the photon-assisted synthesis for 0.5 wt.% of Ru.

Figure 1 shows the disappearance with time under illumination of both Ru metallic precursors using TiO_2_ P25 as semi-conductor host support. First, photolysis of the ruthenium precursors could be neglected under solar light in our experimental conditions, since no disappearance of the main absorption peaks that were assigned to both precursors was observed whatever the precursor used (not shown). The evolution with time of the relative concentration evidenced that both the Ru concentration and the Ru precursor nature are strongly influencing the kinetics of the photocatalytic degradation of the Ru(acac)_3_ and of the RuCl_3_ species at the surface of the irradiated TiO_2_ support. Whatever the Ru content, a slower degradation was observed with the acetylacetonate as compared to the chloride precursor. Indeed, at a content of 0.5 wt.%, a reaction time greater than 200 min was needed for achieving the complete disappearance of the Ru precursor using acetylacetonate, while only 100 min was necessary while using the chloride precursor. Table 1 shows the real metal content of selected Ru/TiO_2_ catalysts after the photon-assisted synthesis, with close agreement being observed between the measured Ru content and the theoretical one derived from the UV-vis absorbance spectra evolution.

For both Ru precursors, increasing the Ru concentration led to increase the necessary reaction time, the effect being more pronounced using acetylacetonate. Indeed, targeting a Ru content of 2 wt.% led only to a photodeposition yield of 35%, where no photodeposition was observed for a Ru concentration of 5 wt.%. Even in the case of the chloride precursor, for which faster kinetics have been observed, the preparation of highly loaded Ru/TiO_2_ catalyst with loadings that are higher than 2 wt.% could not be achieved within a reasonable time under irradiation.

### 3.2. Characterization of the Ru(0.5 wt.%)/TiO_2_ Catalysts

**Figure 2:**
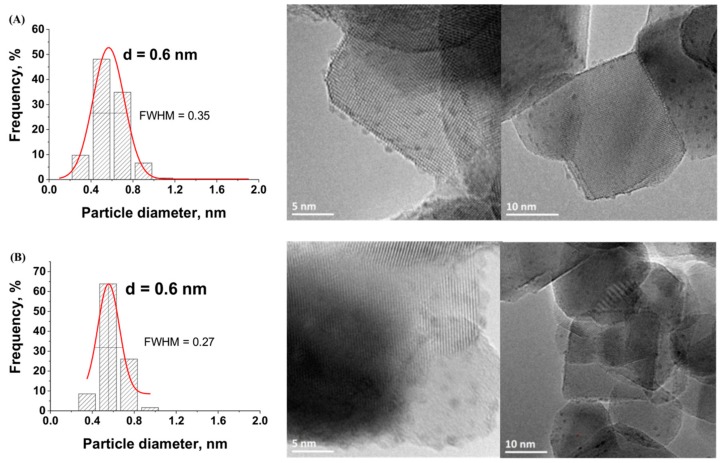
Transmission electron microscopy (TEM) images with the derived histograms of the Ru nanoparticle size distribution for the Ru(0.5 wt.%)/TiO_2_-P25 catalysts prepared from (**A**) acac and (**B**) chloride.

TEM images with the derived histograms of the Ru nanoparticle size distribution for Ru/TiO_2_ catalysts prepared with both Ru(acac)_3_ and RuCl_3_ metallic precursors with a Ru concentration of 0.5 wt.% are shown in Figure 2.

Whether the chloride or the acetylacetonate form of the Ru metallic precursor was used, the nanoparticles that were synthesized on the TiO_2_ support were dispersed homogeneously and no Ru nanoparticle aggregates were observed. Although both precursors strongly differ in terms of chemical nature, small Ru nanoparticles were synthesized on the TiO_2_ support with a similar sharp sub-nanometric particle size distribution centered on 0.6 nm in both cases. A slightly sharper nanoparticle size distribution was obtained using the chloride precursor as compared to that obtained with the acetylacetonate counterpart, with a Full Width at Half Maximum (FWHM) of 0.27 vs. 0.35.

**Figure 3:**
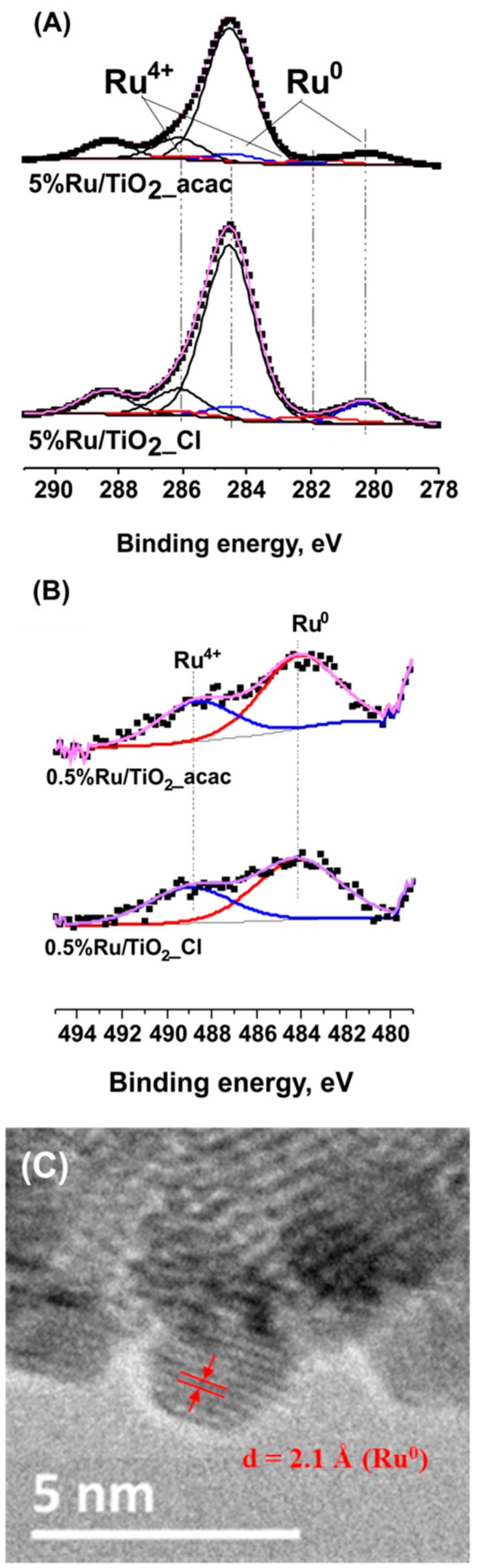
(**A**) Ru 3p and (**B**) Ru 3d + C 1s XPS profile of the Ru(0.5 wt.%)/TiO_2_-P25 catalyst [31]. The carbon C 1s spectra was fitted with major contribution at 284.6 eV corresponding to graphitic sp^2^ carbon and contributions attributed to oxygenated surface groups; (**C**) TEM image of Ru(0.5 wt.%)/TiO_2_-P25 with the measured interplane distance of (101) planes of metallic Ru.

XPS surface characterization and high resolution TEM analysis confirmed the metallic nature of the Ru nanoparticles that were synthesized on the TiO_2_ support (Figure 3). Whatever the Ru precursor used, contributions attributed to both metallic Ru^0^ (484.1 eV) and Ru^4+^ (488.7 eV) species were observed in the Ru 3p_1/2_ orbital XPS spectra of Ru(0.5 wt.%)/TiO_2_ [28]. Within a more complex multi-contribution envelope resulting from the binding energy overlap between Ru 3d and C 1s XPS spectra, the Ru 3d spectra confirmed the presence of both Ru^0^ and Ru^4+^ species, with the presence of two Ru 3d_5/2_-Ru 3d_3/2_ orbital doublet contributions at 280.2 eV and 281.9 eV with a 4.1 eV spin orbit splitting, in addition to the contributions due to the adventious carbon [28].

It was noteworthy that both the ratio of atomic concentrations between Ru^0^ and Ru^4+^ (estimated at 70/30 ± 7 by combining both Ru 3d and Ru 3p spectra results) and the Ru/Ti surface atomic ratio calculated at ca. 0.02 were not influenced by the kind of precursor used. This confirmed that both Ru nanoparticle size distribution and the Ru oxidation state at the TiO_2_ surface were not affected by the choice of the metallic precursor.

In addition, an interplane distance of 2.1 Å corresponding to the (101) atomic planes of metallic Ru was measured on TEM images [29]. The mean Ru nanoparticle size derived from TEM images being of 0.6 nm, the Ru nanoparticles that were supported on TiO_2_ could be considered in a first approximation as being composed in average of only about 3–4 atomic layers. Considering the Ru^0^/Ru^4+^ surface atomic ratio, we propose that the Ru^4+^ species related to the presence of one monolayer resulting from a natural surface oxidation of the metallic Ru nanoparticle, as observed for many supported noble metals [30]. No presence of any residual chlorine was observed by XPS at the surface of TiO_2_ when the chloride precursor was used (Cl 2p XPS spectra not shown).

The different behavior in terms of degradation kinetics observed on TiO_2_ depending on the nature of the Ru metallic precursor used led to propose that the photon-assisted synthesis of Ru nanoparticles on TiO_2_ occurred via two different mechanisms, as it was supported by a previous mechanistic study [31].

In the case of the RuCl_3_ precursor, the photogenerated holes would not be involved in the mechanism proposed, which would only use the photogenerated electrons for obtaining the reduced metallic form of Cu, as the RuCl_3_ precursor is present in aqueous solution as a mixture of Ru chloro-complexes [32,33,34]. By contrast, the mechanism that is proposed in the case of the acetylacetonate precursor involves both holes and electrons charge carriers in the oxidation and reduction steps, respectively, and is derived from the study of Naya et al. on the photon-assisted synthesis of Cu nanoparticles supported on BiVO_4_ photocatalysts from Cu acetylacetonate [34] and schematized in Equations (1)–(4).

The Ru acetylacetonate adsorbed at the TiO_2_ surface can be oxidized by OH° hydroxyl radicals formed via the oxidation of adsorbed water or of surface –OH groups by the photogenerated holes from the valence band, or directly by the holes. Although solar light was used as incident light, only its UV-A fraction activated the Aeroxide^©^ P25 TiO_2_ support, and consequently generated the electron/hole pairs, due to the 3.2 eV band gap energy of the main anatase phase of the material.

(1) $${\mathrm{TiO}}_{2}+\mathrm{Ru}{\left(\mathrm{acac}\right)}_{3}↔\mathrm{Ru}{\left(\mathrm{acac}\right)}_{3\mathrm{ad}}-{\mathrm{TiO}}_{2}$$

(2) $${\mathrm{TiO}}_{2}+h\nu \to e^{-}+h^{+}\left(\mathrm{UVA}\;\mathrm{fraction}\;\mathrm{of}\;\mathrm{the}\;\mathrm{solar}\;\mathrm{light}\right)$$

(3) $$\mathrm{Ru}{\left(\mathrm{acac}\right)}_{3\mathrm{ad}}+3h^{+}\to {\mathrm{Ru}}^{3+}+{\left(\mathrm{acac}\right)}_{\mathrm{ox}}+H^{+}$$

(4) $$\;{\mathrm{Ru}}^{3+}+3e^{-}\to {\mathrm{Ru}}^{0}$$

**Figure 4:**
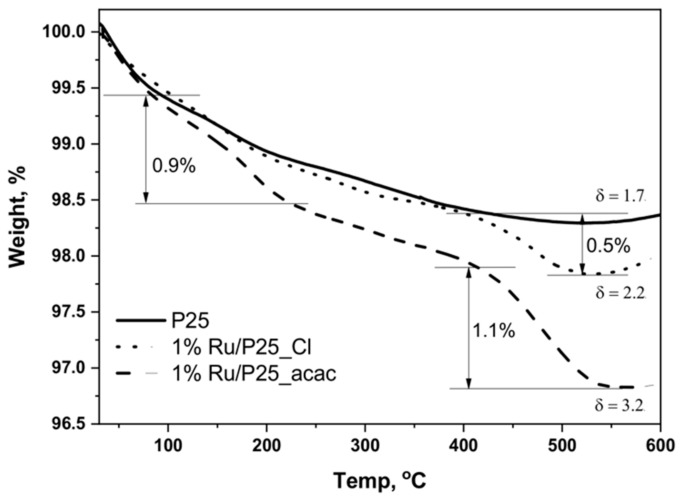
Thermogravimetric analysis (TGA) profiles of TiO_2_ P25 and 1%Ru/TiO_2_ materials prepared from Ru(acac)_3_ and hydrated RuCl_3_ precursors. The weight loss observed on the reference TiO_2_ sample, corresponded to the desorption of molecularly adsorbed water at low temperature and to the surface dehydroxylation (dehydration) at higher temperature. The additional weight loss recorded on the 1%Ru/TiO_2_ materials prepared from RuCl_3_, corresponded to the combustion of carbonaceous residues issued from the partial oxidation of methanol.

The ligand oxidation would generate adsorbed Ru^3+^ ions that can further be reduced into metallic Ru by the photogenerated electrons from the conduction band. As far as Cu was concerned, the direct reduction of Cu(acac)_2_ into metallic Cu was reported to be strongly unfavored as compared to that of Cu^2+^ [35]. Total Organic Carbon measurements showed that the acetylacetonate ligand was mineralized into CO_2_ with a 35% yield at the end of the photo-assisted synthesis process in the case of a 0.5 wt.% Ru content. It was proposed that the acetylacetonate ligand oxidation might follow a usual oxidation pathway for carboned molecules, with consecutive oxidation steps leading to form first partially-oxidized carboned molecules and further short-chain acids, and progressively allowing the complete substrate mineralization into CO_2_ to be achieved. The presence of small amounts of carbon-containing reaction intermediate species at the surface of the Ru/TiO_2_ resulting from the ligand oxidation was confirmed by TGA analysis, with a higher weight loss that is attributed to the desorption and the combustion of those species when using acetylacetonate as compared to that observed on the material prepared with chloride (Figure 4).

However, when considering the presence at the TiO_2_ surface of oxidative species in addition to the photogenerated electrons, we could not fully rule out that the Ru^4+^ surface monolayer observed was formed during the synthesis via the in situ oxidation of Ru^0^ by the photogenerated holes from the valence band or by hydroxyl radicals.

### 3.3. Influence of the Precursor Solution pH and Preparation of the Ru(5 wt.%)/TiO_2_ Catalyst

**Figure 5:**
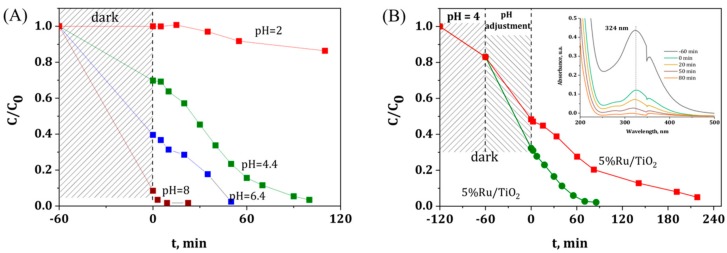
(**A**) Influence of the solution pH on the disappearance of the RuCl_3_ precursor in the presence of TiO_2_-P25 as a function of the illumination time for a Ru content of 0.5 wt.%. (**B**) Disappearance of the RuCl_3_ precursor in the presence of TiO_2_-P25 as a function of the illumination time at pH = 6.4 and pH = 8.0, with a Ru content of 5 wt.%. Inset: UV-vis absorbance spectra evolution as a function of time during the photon-assisted synthesis of Ru(5 wt.%)/TiO_2_ at pH = 8.

Figure 5A shows the influence of the pH of the aqueous Ru precursor solution on the photodeposition kinetics of 0.5 wt.% Ru on TiO_2_. It was worth noting that increasing the pH led to significantly enhancing the adsorption of the Ru precursor species on the TiO_2_ support in the dark, while reducing strongly the time necessary for a complete precursor disappearance, and consequently for preparing the Ru(0.5 wt.%)/TiO_2_ material. This behavior with increasing the pH of the solution has been attributed to the amphoteric nature of the TiO_2_ support, for which the surface is negatively-charged for pH higher that the zero-charge point (i.e., ca. 6.25 for TiO_2_-P25), and positively-charged for pH lower that the zero-charge point [36]. Thus, the TiO_2_ surface is considered to be negatively-charged when the photodeposition was performed at pH = 8, whereas the surface charge increased gradually with decreasing the solution pH, so that the reaction was implemented on a zero charge surface at pH 6.4 and on a positively-charged surface at pH = 2.

In water, RuCl_3_ species are very complex systems and co-exists in the form of various aqueous chloro-complexes, for which the thermodynamic equilibrium depends on parameters, like the pH, temperature, and the concentration [32]. The determination of Ru forms in solution remains however very challenging, due to the multiple degrees of polymerization of the Ru chloro complexes, as well as the co-existence of different oxidation states for Ru [37]. Considering the aqueous Ru chloro-complexes distribution diagrams with the chloride concentration evolution, we could propose to rule out the existence of negatively-charged Ru complexes like RuCl_6_^3−^, RuCl_5_(H_2_O)^2−^ and RuCl_4_(H_2_O)_2_^−^ in the TiO_2_ suspension, and we assumed that the Ru species are most probably present as a mixture of RuCl_3_(H_2_O)_3_, and of positively-charged RuCl_2_(H_2_O)_4_^+^ and RuCl(H_2_O)_5_^2+^ species [33,34].

As a result, at low pH, the adsorption of Ru species on the positively charged TiO_2_ surface was unfavored due to electrostatic repulsion, while by contrast the increase in the solution pH above the isoelectrical point strongly enhanced the adsorption of Ru species on the positively charged TiO_2_ surface.

The strong influence of the pH on the adsorption behavior and consequently on the photodeposition kinetics opened the possibility of preparing Ru/TiO_2_ catalysts with a high Ru metal loading of 5 wt.%, as shown in Figure 5B and in Table 1. At a higher Ru loading, a similar pattern was obtained, with a higher adsorption on the TiO_2_ support at pH 8 as compared to that observed at pH 6.4, so that a reaction time greater than 200 min was needed for achieving the complete precursor disappearance at pH 6.4, while only 90 min was necessary at pH 8.

### 3.4. Characterization of the Ru(5 wt.%)/TiO_2_ Catalyst

**Figure 6:**
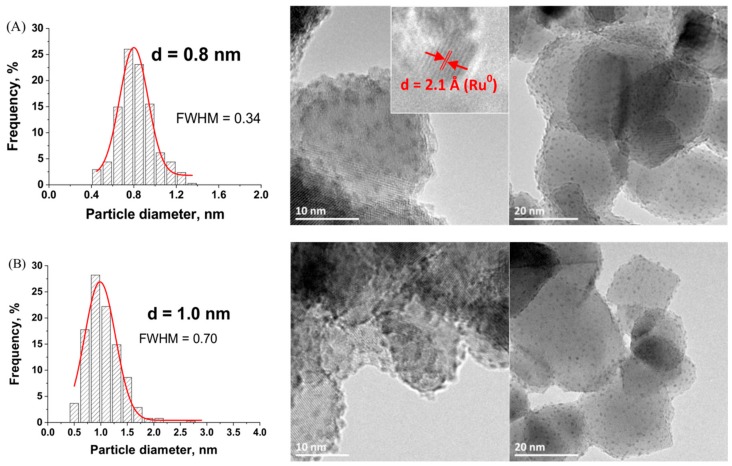
TEM image and the Ru nanoparticle size distribution for the Ru(5wt.%)/TiO_2_-P25 catalysts prepared (**A**) at pH = 8,0 with the measured interplane distance of (101) planes of metallic Ru; and (**B**) pH = 6.4.

Figure 6 shows the TEM images and the derived histograms of the Ru nanoparticle size distribution for the Ru(5 wt.%)/TiO_2_ catalyst prepared at pH = 8.0 and pH = 6.4. First, in both cases the supported nanoparticles were dispersed homogeneously and no Ru aggregates were observed. Small and sharp nanoparticle size distributions were obtained, centered on 0.8 nm (FWHM = 0.34 nm) and 1.0 nm (FWHM = 0.70 nm) at pH = 8,0 and pH = 6.4, respectively. The slightly smaller and sharper particle size distribution achieved at pH = 8.0 was attributed to the higher adsorption of the aqueous Ru chloro-complexes with the negatively-charged TiO_2_ surface at this pH, which led to maintain a higher and more narrow dispersion at the support surface. When performing the reaction at pH 8, it is remarkable that the increase in the Ru content by an order of magnitude did not result neither in a strong increase in the average particle size, nor in its broadening.

**Figure 7:**
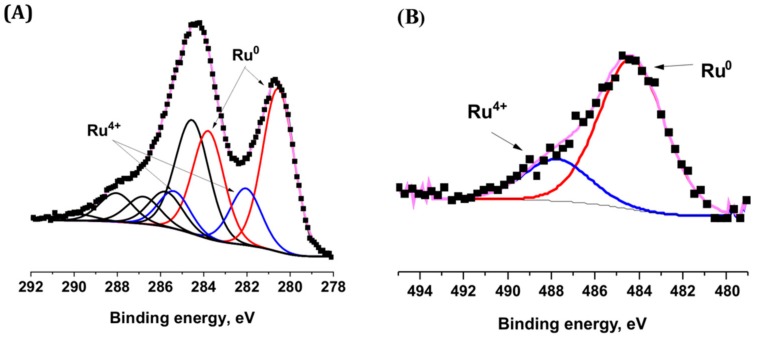
(**A**) Ru 3d + C 1s; (**B**) Ru 3p XPS profile of the Ru(5 wt.%)/TiO_2_-P25 catalyst. No presence of any residual chlorine was observed by XPS at the surface of TiO_2_ when the chloride precursor was used (Cl 2p XPS spectra not shown).

The metallic nature of the supported nanoparticles of the Ru(5 wt.%)/TiO_2_ catalyst was confirmed by the high resolution TEM image (insert of Figure 6A) that evidenced an interplane distance of 2.1 Å corresponding to the (101) atomic planes of metallic Ru, as well as by XPS surface characterization (Figure 7). The Ru 3p_1/2_ and Ru 3d_5/2_ − Ru 3d_3/2_ orbital XPS spectra exhibited similar patterns than those that were recorded on the TiO_2_ support with a low 0.5 wt.% of Ru. The Ru^0^/Ru^4+^ atomic concentration ratio was estimated at 80/20 ± 7. The XPS patterns differed only in terms of intensity of both Ru^0^ and Ru^4+^ doublets for the 3d orbitals in respect to the C1s main peak at 284.6 eV attributed to contamination carbon, and consequently in terms of Ru/Ti surface atomic ratio. The surface atomic ratio was calculated at 0.23 for 5 wt.% of Ru vs. 0.02 for 0.5 wt.% of Ru. The approximatively ten-fold higher ratio while increasing the Ru loading by an order of magnitude, characterized the maintain of a very high dispersion of small size Ru nanoparticles at the TiO_2_ support surface confirmed the slight increase in mean particle size from 0.6 nm to 0.8 nm and the slight increase in FHWM from 0.27 nm to 0.34 nm.

**Figure 8:**
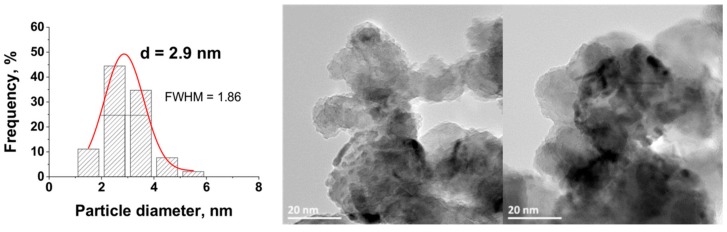
TEM image and the Ru nanoparticle size distribution for the Ru(5 wt.%)/TiO_2_-P25 catalyst prepared via classical impregnation with final reduction in hydrogen at 200 °C.

This highlighted the interest of the synthesis method for preparing Ru/TiO_2_ catalysts with well-calibrated Ru nanoparticles dispersed at the surface of the TiO_2_ support. This contrasted with the Ru(5 wt.%)/TiO_2_-P25 catalysts prepared via classical impregnation with final reduction under hydrogen at 200 °C, which exhibited a broader nanoparticle size distribution (FHWM = 1.86) centered on a larger average particle size, at 2.9 nm (Figure 8).

### 3.5. Fine Control of the Ru Particle Size Distribution

**Figure 9:**
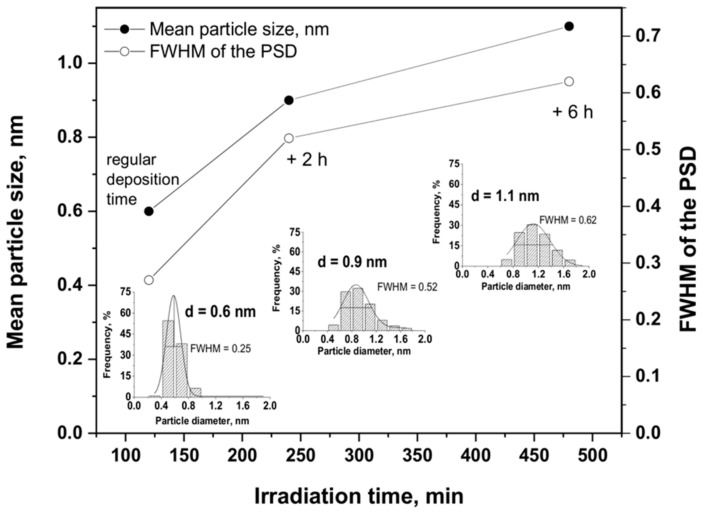
Evolution of the mean Ru particle size and of the distribution Full Width at Half Maximum (FWHM) as a function of the irradiation time for the 1%Ru/TiO_2_ catalyst prepared with the chloride precursor. Inset: Ru particle size distributions obtained for 2 h, 4 h and 8 h of irradiation, corresponding to the regular irradiation time, and 2 h and 6 h of extra-time irradiation in the absence of Ru precursor, respectively.

It has been further demonstrated that a fine monitoring of the metal Ru particle size on the TiO_2_ support was possible via a controlled growth of the Ru nanoclusters under irradiation, which was achieved by extending the duration of the irradiation after the full conversion of the Ru precursor salt was achieved. Indeed, Figure 9 shows that the mean Ru particle size progressively and slightly increased from 0.6 nm to 0.9 nm and further to 1.1 nm, when 2 h and 6 h of supplementary irradiation time was provided to the system, respectively. Simultaneously, the FWHM values slightly increased from 0.25 nm to 0.52 nm and further to 0.62 nm, evidencing a slight broadening of the particle size distribution. Whatever the irradiation time, the nanoparticles remained homogeneously dispersed on the support with a small mean particle size and without the formation of large Ru nanoparticle aggregates. This highlighted the possibility to provide on-demand Ru/TiO_2_ catalysts with a monomodal particle size distribution and a well-calibrated mean particle size for studying and optimizing the reactivity of heterogeneous catalysts in different size-dependent or structure-sensitive reactions [5,6].

### 3.6. Influence of Thermal Reduction

**Figure 10:**
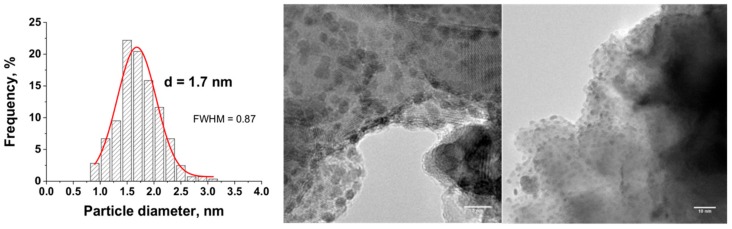
TEM images with the derived histograms of the Ru nanoparticle size distribution for the Ru(5 wt.%)/TiO_2_-P25 catalyst obtained by the photon-assisted preparation method, after treatment at 200 °C under hydrogen for 2 h.

The stability of the Ru(5 wt.%)/TiO_2_ catalyst has been evaluated by submitting the as-prepared catalysts to further reduction under hydrogen at 200 °C for 1 h. Those conditions are classical conditions for getting metallic Ru nanoparticles on supports such as e.g., titania, activated carbon, alumina, or zirconia by classical incipient wetness or wet impregnation with acetylacetonate or chloride precursors [28,36,38]. In addition, many reactions that are involved in the catalytic conversion of biomass-derived molecules on Ru catalysts are also implemented at temperatures lower than 200 °C [20], so that 200 °C remains an appropriate temperature for evaluating the thermal stability of the catalysts. Figure 10 shows the TEM images with the derived histograms of the Ru nanoparticle size distribution for the treated Ru/TiO_2_-P25 catalyst.

It has been evidenced that the Ru(5 wt.%)/TiO_2_ catalyst displayed a good resistance to nanoparticle sintering and growth. Indeed, the mean Ru particle size slightly increased from 0.8 nm to 1.7 nm, with a slight broadening of the distribution from 0.27 nm to 0.87 nm in terms of FWHM values. The nanoparticles remained homogeneously dispersed on the support with a small mean particle size still far lower and a particle size distribution still far sharper to those that were obtained on the Ru(5 wt.%)/TiO_2_ catalyst prepared via classical impregnation with reduction in H_2_ at 200 °C.

## 4. Conclusions

In this study, we proved the effectiveness of an elegant low-temperature one-step photon-assisted synthesis method taking advantage of the redox photoactivity of the TiO_2_ semi-conductor support under solar light, for preparing highly dispersed TiO_2_ supported metallic Ru catalysts that are promising heterogeneous catalysts in different key-reactions that are involved in the catalytic biomass conversion into fuel additives, biofuels, or biochemicals. They exhibited an enhanced catalytic activity in the combined hydrogenation of levulinic acid into γ-valerolactone using formic acid as internal hydrogen source, as well as in the levulinic acid hydrogenation with external hydrogen, in comparison to that shown by the reference counterpart catalysts that were prepared by incipient wet impregnation [39]. This was possible thanks to the stabilization of well-dispersed, small, and uniform metal crystallites.

Far smaller Ru nanoparticles with sharper size distribution were synthesized when compared to the catalysts that were prepared via wet or incipient wetness impregnation with final thermal reduction in hydrogen. While XPS and TEM analyses evidenced the highly dispersed and metallic state of the Ru nanoparticles, we have demonstrated that by implementing a suitable photo-assisted synthesis protocol, the size distribution of the supported sub-nanometric size Ru nanoparticles was only very slightly influenced by the Ru content within a large 0.5–5 wt.% range. Further, a fine monitoring of the metal Ru nanoparticle size on the TiO_2_ support was possible via a controlled growth of the Ru nanoclusters under irradiation. Those catalysts exhibited a good resistance to thermally activated nanoparticle sintering. This opens the possibility to prepare on-demand Ru/TiO_2_ catalysts with a monomodal and well-calibrated particle size distribution for performing fundamental investigation on the reactivity of heterogeneous catalysts in different size-dependent or structure-sensitive reactions.

## Figures and Tables

**Table 1:** Ru content in Ru/TiO_2_ materials determined by inductively coupled plasma optical emission spectroscopy (ICP-OES).

Ruthenium Precursor, pH	Targeted Ru Content, wt.%	Ru Content, wt.% ^a^
acetylacetonate	0.5	0.45
chloride, 4.4	0.5	0.46
chloride, 6.4	1	0.92
chloride, 8.0	5	4.9
chloride, 6.4	5	4.8

^a^ a maximum of about 10% relative difference was observed between the measured Ru content and the theoretical one derived from the UV-vis absorbance profile, demonstrating that the direct monitoring of the Ru precursor disappearance by UV-Vis spectrophotometry was a fast and suitable method for obtaining the Ru content in the catalysts.
